# Supplementary material for: Active site specificity profiling datasets of matrix metalloproteinases (MMPs) 1, 2, 3, 7, 8, 9, 12, 13 and 14
Source: Data Brief. 2016 Feb 22;7:299–310. doi: 10.1016/j.dib.2016.02.036 (PMC4777984; doi:10.1016/j.dib.2016.02.036)
Supplement: Supplementary file 10 — Supplementary material [file mmc10.zip › WebPICS_hMMP2_G_1%/P1prime.html]

 

PICS results


|  |  |
| --- | --- |
| **P1prime\_C**  8 in 246 sites   3.3 %    effects > 10 perc. pnts.  (vice-versa in brackets)  P2prime\_T: 29.4 (11.7) |  |
| **P1prime\_I**  32 in 246 sites   13.0 %    effects > 10 perc. pnts.  (vice-versa in brackets)  P3prime\_G: 16.2 (14.0) |  |
| **P1prime\_M**  10 in 246 sites   4.1 %    effects > 10 perc. pnts.  (vice-versa in brackets)  P2prime\_M: 17.2 (24.5) |  |
| **P1prime\_V**  18 in 246 sites   7.3 %    effects > 10 perc. pnts.  (vice-versa in brackets)  P3\_P: 50.2 (16.8) |  |
| **P1prime\_W**  5 in 246 sites   2.0 %    effects > 10 perc. pnts.  (vice-versa in brackets)  P2prime\_Y: 16.7 (10.5) |  |
